# Interventions for patients with prostate cancer on active surveillance: a narrative review

**DOI:** 10.1111/bju.70005

**Published:** 2025-10-06

**Authors:** Simran Gill, Runzhi Chen, Benjamin W. Lamb, Caroline M. Moore, James W.F. Catto, Jack Cuzick, Peter D. Sasieni, Prabhakar Rajan

**Affiliations:** ^1^ Centre for Cancer Cell and Molecular Biology, Barts Cancer Institute Queen Mary University of London London UK; ^2^ Centre for Cancer Screening, Prevention, and Early Diagnosis, Wolfson Institute of Population Health Queen Mary University of London London UK; ^3^ Department of Urology Barts Health NHS Trust London UK; ^4^ Department of Urology University College London Hospitals NHS Foundation Trust London UK; ^5^ Division of Surgery and Interventional Science University College London London UK; ^6^ Department of Urology Royal Surrey County Hospital NHS Foundation Trust Guildford UK; ^7^ Division of Clinical Medicine, School of Medicine and Population Health University of Sheffield Sheffield UK; ^8^ Department of Urology Sheffield Teaching Hospitals NHS Foundation Trust Sheffield UK

**Keywords:** prostate cancer, active surveillance, hormonal therapy, dietary supplements, exercise

## Abstract

**Objective:**

To examine contemporary evidence supporting non‐surgical interventions for patients with early prostate cancer (PCa) on active surveillance (AS).

**Methods:**

A literature search was conducted using the databases PubMed, Medline, and Embase in January 2024 to identify relevant articles published from 2011 onwards. Randomised controlled trials (RCTs) and cohort studies reporting on interventions in patients with PCa on AS were included.

**Results:**

Several studies have investigated a range of non‐surgical interventions, from exercise and food supplements to androgen receptor pathway inhibitors (ARPIs). The largest RCTs of ARPIs have shown the greatest effect in delaying disease progression to aggressive PCa, however, concerns exist around toxicity, long‐term oncological safety, and mortality benefit. Nutraceuticals, dietary modifications, and exercise appear to be well tolerated, but evidence of oncological benefit from large‐scale RCTs is lacking. A major challenge is the lack of consensus on criteria for entry to and exit from AS as pre‐biopsy magnetic resonance imaging reduces diagnosis of the lowest‐risk disease. Molecular and imaging biomarkers may help refine baseline risk stratification and guide monitoring during surveillance.

**Conclusion:**

Current evidence supporting interventions for patients on AS remains largely based on small prospective cohort studies or open‐label phase II trials. There is growing interest in short‐term ARPIs for patients at higher risk of disease progression, while nutraceuticals, diet and exercise may have a role for long‐term use in lower‐risk patients. Large, well‐powered RCTs with long‐term outcomes testing different interventions and incorporating molecular and imaging biomarkers are warranted.

**Abbreviations**
5ARI: 5α‐reductase inhibitor
AR: androgen receptor
ARPI: androgen receptor pathway inhibitor
AS: active surveillance
CPG: Cambridge Prognostic Group
DHT: dihydrotestosterone
GS: Gleason score
HR: hazard ratio
mpMRI: multiparametric
NICE: National Institute for Health and Care Excellence
PCa: prostate cancer
RCT: randomised controlled trial
RP: radical prostatectomy
RT: radiotherapy

## Introduction

Prostate cancer (PCa) is the most frequently diagnosed malignancy in men in the UK, accounting for 28% of all new male cancers (>55 000 cases/year), and the second leading cause of mortality [1]. The incidence is predicted to double over the next ∼10–15 years, driven largely by an aging population [2]. Approximately 40% of cases present with early, localised disease, which carries an excellent prognosis with either active treatment or monitoring [1, 3, 4]. Diagnosis typically follows a raised PSA level or an abnormal DRE, prompting multiparametric MRI (mpMRI) and biopsy.

Risk stratification of non‐metastatic PCa [5, 6] has traditionally been based on PSA levels, DRE findings, and biopsy‐derived Gleason score (GS) [7] (Table S1). Conventional risk stratification relies on disease burden estimates from a standard number of systematic biopsies taken at predetermined sites [8]. However, in MRI‐targeted biopsies, lesion‐focused sampling can lead to ‘oversampling’ [9], hence maximum cancer core length and highest GS from targeted cores may provide more accurate cancer burden estimates [9, 10].

The UK National Institute for Health and Care Excellence (NICE) now recommends risk stratification into the five‐tiered Cambridge Prognostic Groups (CPGs; Table S1) based on PCa‐specific mortality [6, 11]. The CPG system helps identify patients who are suitable for active surveillance (AS) to delay or avoid curative treatment‐related side effects through active disease monitoring [12]. Since those within CPG2 and CPG1 have similar disease progression rates with conservative management [13], NICE equally recommends either AS, radical prostatectomy (RP), or radiotherapy (RT) for both groups.

The Prostate Testing for Cancer and Treatment (ProtecT) trial randomised patients with screen‐detected PCa to active monitoring, RP or RT [4]. Active monitoring involved PSA testing every 3 months in Year 1, then every 6–12 months, with review if PSA rose by ≥50% [14] – a less intensive schedule than current protocols. After 15 years, mortality was similar across all arms, but metastases were more frequent with active monitoring (9.4% vs 4.7% [RP] and 5.0% [RT]) [4]. These findings suggest treatment may benefit some patients with PSA screen‐detected PCa, although PSA and standard biopsy may not optimally identify those who would benefit.

In the 2024 UK National Prostate Cancer Audit, only 8% of men in England with localised low‐risk PCa received radical treatment, although rates reached 29% in some centres [3]. A frequent reason for discontinuing AS without disease progression is patient or clinician anxiety about progression or PSA changes [15]. Discontinuation rates vary widely – from <1% at 6 years with an MRI‐led approach and no protocol‐based biopsy [16], to 25% in a centre performing repeat transperineal mapping biopsies [17].

There is growing interest in minimally toxic non‐surgical strategies to delay or prevent disease progression in patients on AS. The intensity of an approach should align with progression risk – for example, dietary, lifestyle, and nutraceutical interventions may suit those at lowest risk, while higher‐risk patients might consider pharmaceutical options. This review summarises the current evidence on non‐surgical interventions for patients with localised PCa on AS and outlines priorities for future research.

## Hormonal Therapies

Prostate tumourigenesis and disease progression are largely driven by androgens binding to the androgen receptor (AR) [18]. Testosterone, the predominant male androgen, is synthesised by Leydig cells of the testis under the control of the hypothalamic–pituitary–gonadal axis and subsequently converted to dihydrotestosterone (DHT) by 5α‐reductase types 1 and 2. 5α‐reductase inhibitors (5ARIs; finasteride and dutasteride) are established treatments for BPH, reducing DHT production, prostate volume, and urinary symptoms associated with an enlarged prostate [19]. Notably, 5α‐reductase type 1 expression is increased in localised PCa [20, 21], highlighting its potential as a therapeutic target. Second‐generation AR pathway inhibitors (ARPIs), including non‐steroidal competitive antagonists such as enzalutamide, apalutamide and darolutamide, achieve more potent AR blockade than earlier agents (e.g., bicalutamide and flutamide) and have demonstrated survival benefits in metastatic disease [22, 23]. Given the central role of AR signalling across the disease spectrum, pharmacological suppression of androgen biosynthesis and downstream AR activity has been explored as a strategy to delay progression in men on AS (Table 1).

**Table 1:** Summary of interventional clinical trials which investigated hormonal agents as interventions in men with low‐ or intermediate‐risk prostate cancer on active surveillance.

Authors/year	Study design	Number of patients	Population	Intervention	Primary endpoint	Follow‐up period	Conclusions
Fleshner et al. [24] 2012	Prospective randomised double‐blind placebo‐controlled trial (REDEEM)	302	T1c–T2a PSA ≤11 ng/mL GS ≤6 Diagnostic biopsy where <4 of 10 positive cores and <50% of any one core positive or saturation biopsy where 2–3 of 20 positive cores and <50% of any one core positive	Once‐daily dutasteride 0.5 mg for 3 years	Time to disease progression; either pathological (GS ≥4, four or more cores involved, ≥50% core involvement) or therapeutic progression	3 years	Dutasteride: 38% Placebo: 48% HR 0.62 (95% CI 0.43–0.89), *P* = 0.009
Moore et al. [27] 2017	Prospective randomised double‐blind placebo‐controlled trial (MAPPED)	42	GS <7 (3 + 4) PSA <15 ng/mL >0.2 mL lesion on T2‐weighted mpMRI Confirmed PCa on biopsy	Once‐daily dutasteride 0.5 mg for 6 months	Percent reduction in PCa lesion volume	6 months	Dutasteride: 36% average reduction Placebo: 12% average increase Difference in percent reduction 48% (95% CI 27.4–68.3), *P* < 0.0001
Shore et al. [32] 2022	Prospective randomised open‐label trial (ENACT)	227 Low risk (*n* = 121) Intermediate risk (*n* = 106)	Defined per National Comprehensive Cancer Network guidelines Low‐risk PCa: T1c–T2a, N0, M0 PSA <10 ng/mL GS <6 ECOG PS <2 Intermediate‐risk PCa: T2b–T2c, N0, M0 PSA <20 ng/mL GS <7 (3 + 4 only) ECOG PS <2	160 mg enzalutamide monotherapy for 1 year	Time to disease progression; either pathological (>1 increase in Gleason pattern or >15% increase in positive cores) or therapeutic progression (secondary treatment)	Up to 2 years	Enzalutamide 46% risk reduction in disease progression compared to AS alone HR 0.54 (95% CI 0.33–0.89, *P* = 0.02)
Barrett et al. [34] 2022	Prospective open‐label single‐arm phase II feasibility trial (TAPS01)	9	CPG1‐2 classification (low or favourable intermediate risk)	Once‐daily apalutamide 240 mg for 90 days	TV, GV, TV/GV ratio measured by mpMRI at Day 90 and at 6‐ and 18‐month follow‐up	90 days Further follow‐up at 6 months and 18 months	Day 90 median percentage reduction (all statistically significant, *P* < 0.0001): TV –54.2% (range −74.1% to −13.8%) GV –38.2% (range −51.8% to −23.5%) TV/GV –27.2% (range −61.5% to −7.5%) 6 months: TV –31.9%, *P* = 0.0007 GV returned to baseline TV/GV −28.7%, *P* = 0.0009 18 months: Sustained both TV and TV/GV reduction (−18% and −23.8% respectively, *P* = 0.01)
Schweizer et al. [33] 2023	Prospective open‐label single‐arm phase II clinical trial	22 GG 1 (*n* = 15) GG 2 (*n* = 7)	Very low risk PCa: T1c PSA <0.15 ng/mL GS 6 <2 core biopsies with <50% cancer of biopsy core or unilateral disease with any % involvement Low‐risk PCa: <T2a PSA <15 ng/mL GS 6 Low‐ to intermediate‐risk PCa: T1c PSA <15 ng/mL GS 3 + 4 present in <50% of one core GS 6 across all other cores	Once‐daily apalutamide 240 mg for 90 days	Percentage of patients with a negative repeat biopsy at the end of study (Day 90)	90 days	59% of patients had residual cancer on post‐treatment biopsy Median time to first positive biopsy was 364 days (95% CI 91–742 days)
Cumberbatch et al. [28] Ongoing Expected 2027	Prospective open‐label randomised controlled phase III trial (FINESSE)	Ongoing (estimated 550)	Histopathological diagnosis of low‐ or intermediate‐risk adenocarcinoma Gleason GG ≤2 Radiological stage ≤T2c cN0 cM0 as defined by bpMRI/mpMRI imaging PSA ≤20 ng/mL PSA density ≤0.2 ng/mL Biopsy criteria: Maximum cancer core length is ≤10 mm ≤3 cores involved with cancer	Once‐daily finasteride 5 mg for 2 years	Adherence to AS at 2 and 5 years after diagnosis, defined as absence of change in treatment to radical therapy or treatment of advanced disease	4 years	Trial ongoing
Gnanapragasam et al. [35] Ongoing Expected 2031	Prospective randomised controlled phase III trial (TAPS02)	Ongoing (estimated 402)	CPG2 CPG1 with PSA high density >0.15 and: Likert or PI‐RADS 4/5 lesion of ≥10 mm size ≥50% biopsy core involvement (number of positive cores/all cores taken) with target biopsies counted as one if Likert or PI‐RADS 3 lesion	Once‐daily apalutamide 240 mg for either 3 or 6 months	Reduction in MRI‐defined tumour volume at 12 months Disease progression ≥GG 3 or T3 stage and composite score of ≥CPG3 disease at 3 years	12 months after end of treatment Further follow‐up at 3 years	Trial ongoing

AS, active surveillance; bpMRI, biparametric MRI; CPG, Cambridge Prognostic Group classification; ECOG PS, Eastern Cooperative Oncology Group performance status; GG, Grade Group; GS, Gleason score; GV, gland volume; HR, hazard ratio; mpMRI, multiparametric MRI; PCa, prostate cancer; PI‐RADS, Prostate Imaging–Reporting and Data System; TV, tumour volume; TV/GV, tumour volume/gland volume ratio.

### 5α‐Reductase Inhibitors

In the Reduction by Dutasteride of Clinical Progression Events in Expectant Management (REDEEM) trial, 302 patients with low‐risk PCa (low‐volume GS 5–6) on AS were randomised to receive dutasteride or placebo to investigate the safety and efficacy of dutasteride on disease progression, defined as pathological or therapeutic progression (at least one of the following: >4 cores involved; >50% of any one core involved; or a GS >4) or therapeutic progression (RP, RT or hormonal therapy) [24]. At 3‐year follow‐up, patients treated with dutasteride had a significant risk reduction of disease progression compared to placebo‐treated men (38% vs 48% of men with disease progression respectively; hazard ratio [HR] 0.68, 95% CI 0.43–0.89; *P* = 0.009) [24]. No patient developed metastatic disease during follow‐up, although the authors acknowledged that larger trials of longer duration are required.

The rate of disease progression in the REDEEM trial was higher than rates seen in similar surveillance studies [25, 26], which the authors attributed to potential methodological differences in patient criteria or biopsy sampling due to shrinkage of prostate tissue secondary to dutasteride and improved detection of higher‐grade cancer in a smaller prostate [24]. There were similar overall rates of adverse events in the two groups; in particular, 21.7% of dutasteride patients compared to 14.2% of placebo patients experienced adverse events related to sexual function (impotence, decreased libido, ejaculation disorders) [24].

In a similar study, the MRI in Primary Prostate Cancer after Exposure to Dutasteride (MAPPED) trial, 42 men with low‐ and intermediate‐risk PCa were randomised to receive dutasteride or placebo for 6 months to evaluate the effect of dutasteride on MRI‐estimated tumour volume [27]. A significant reduction in lesion volume from baseline was observed in the dutasteride group compared to the placebo group after 6 months (36% reduction vs 12% increase; *P* < 0.0001) [27]. Notably, histological upgrading to Gleason >4 + 3 on exit biopsy was reported in only 20% (3/15) of patients on dutasteride compared to 46% (6/13) on placebo [27]. The rates of upgrading were high in both the dutasteride and placebo groups due to untargeted biopsies at baseline. There was a deterioration in sexual function measured by the International Index of Erectile Function (IIEF‐15) in 25% of men on dutasteride compared to 16% of men on placebo, but no change in drop‐out rate.

The open‐label Finasteride Evaluation in Surveillance for Prostate Cancer (FINESSE) study is currently recruiting patients to a prospective randomised controlled trial (RCT) of AS alone vs AS with 2 years of finasteride (5 mg once a day), with a planned follow‐up period of 4 years [28]. The surveillance protocol includes PSA testing, mpMRI, and biopsy as per standard practice, and will also assess tolerability and quality‐of‐life measures. The primary endpoint is adherence to AS (surveillance could cease due to treatment by RP, RT or palliation for metastatic PCa).

In summary, 5ARIs show promise for AS patients, although evidence is limited by the absence of large, adequately powered trials. Initial concerns from chemoprevention studies about increased high‐grade tumours [29] have been attributed to improved biopsy detection in prostates reduced in size by treatment, with similar rates of high‐grade tumours at RP in both arms [30]. Although 5ARIs are not licensed for PCa risk reduction, they are widely prescribed in men with larger prostates and urinary tract symptoms without the need to exclude PCa before initiation. In men on 5ARIs undergoing AS, PSA levels should be doubled to estimate the true PSA, and PSA kinetics (including doubling time) should be closely monitored, as rises or rapid doubling may indicate progression. Evidence from chemoprevention studies indicates that men with larger prostates experience a marked reduction in PCa incidence, particularly low‐grade tumours, while those with LUTS benefit from improved urinary flow and symptom control [31]. These findings suggest 5ARIs merit further study in these AS subgroups.

### Androgen Receptor Pathway Inhibitors

The ENACT trial evaluated the efficacy and safety of enzalutamide in men on AS by randomising 227 patients to either AS plus enzalutamide monotherapy or standard AS with up to 2 years of follow‐up [32]. Patients were histologically proven to have low‐ or intermediate‐risk localised PCa and stratified by cancer risk (low vs intermediate) and biopsy type (mpMRI‐targeted or non‐mpMRI‐targeted) [32]. There was a significant 9.1% absolute risk reduction in disease progression, defined either pathologically (increase in Gleason pattern or higher proportion of cancer‐positive cores) or therapeutically (radical treatment), in the enzalutamide group compared to standalone AS (HR 0.54, 95% CI 0.33–0.89; *P* = 0.02) [32]. Similarly, time to PSA progression (defined as the duration between initial PSA testing and a secondary rise in serum PSA levels of 25% or more of the baseline or nadir, or an absolute increase of ≥2 ng/mL) was significantly delayed by 6 months with enzalutamide treatment compared to AS (HR 0.71, 95% CI 0.53–0.97; *P* = 0.03) but this effect was not observed at 2 years [32]. Some doubt the clinical relevance of these definitions of PSA progression, when it is expected that the PSA will rise in line with prostate growth during AS.

Two phase II clinical trials have reported on tumour response of apalutamide in men on AS measured by either negative biopsy [33] or mpMRI parameters [34]. Schweizer et al. [33] conducted an open‐label, single‐arm prospective cohort study in 22 patients with very‐low‐ to favourable intermediate‐risk PCa (as per the National Comprehensive Cancer Network criteria) who received 90 days of apalutamide, with the primary endpoint of negative prostate biopsy immediately following treatment. Based on a per‐protocol analysis, 59% of patients demonstrated negative residual cancer on biopsy (*n* = 22 analysed; HR 0.59, 95% CI 0.36–0.79; *P* < 0.001) [33]. Barrett et al. [34] also conducted a phase II clinical trial of nine men on AS with CPG1 and CPG2 disease treated with apalutamide for 90 days, and assessed tumour response using mpMRI after 90 days of treatment. In this study, there was significant median percentage reduction in both gland volume (38.2%) and tumour volume (54.2%) at 90 days (*P* < 0.0001). Moreover, the effect on tumour volume was maintained in tumour volume at 6 and 18 months despite gland volume returning to baseline at 6 months. The conclusions drawn from the results of both studies are limited by the small sample sizes.

The multicentre Therapeutics in Active Prostate cancer Surveillance 2 (TAPS02) RCT is currently recruiting patients to investigate the effect of short‐term apalutamide vs placebo in men at high risk of disease progression on AS [35]. A primary endpoint of the first phase is to observe a 20%–50% reduction in mpMRI‐defined tumour volume at 12 months following intervention in either of the apalutamide arms, with the most effective dosage taken forward to phase 2 [35]. The primary endpoint for phase 2 is progression to CPG3 disease, with other endpoints including trial acceptability, patient‐reported outcomes, and quality‐of‐life metrics [35].

A major opposition to ARPI use is unwarranted side effects in AS patients who are otherwise unaffected by their disease. In the ENACT trial, 55.4%, 36.6% and 30.4% of enzalutamide‐treated patients reported fatigue, gynaecomastia and nipple pain, respectively, compared to 3.5%, 1.8% and 0% of patients on AS alone [32]. Erectile dysfunction and decreased libido were also more common (17.9% and 8.0% with ARPI vs 1.8% and 0.9%, respectively). Sexual and physical function declined during treatment but recovered within 24 months of cessation [32].

In the apalutamide studies, both fatigue and breast pain or gynaecomastia were the main side effects. Schweizer et al. [33] reported grade 1 fatigue and gynaecomastia in 70% of patients, while Barrett et al. [34] observed that all 11 patients reported at least one adverse event, with the most common events reported as fatigue (55.5%), rash (44.4%), and breast pain (44.4%). Barrett et al. [34] observed a reduction in global health status, physical, role and social functioning (assessed by European Organisation for Research and Treatment of Cancer [EORTC] QLQ‐C30) between baseline and Day 90, which began to recover 6 weeks post‐treatment. Emotional and cognitive functioning, and global health (assessed by the EuroQol Visual Analogue Scale [EQ‐VAS]) remained stable. No patients discontinued treatment and all side effects had resolved or were resolving at 6 weeks [34]. Gravis et al. [36] conducted a phase II trial to assess the safety and quality‐of‐life impact of 1 year of apalutamide treatment compared to standalone AS in patients with low‐ or intermediate‐risk PCa. Quality‐of‐life scores (measured by the 12‐item Short‐Form Health Survey [SF‐12]) were comparable between the two groups at 6 and 12 months, indicating no significant negative impact on quality of life during or after treatment [36].

In summary, ARPIs show potential for AS patients. However, the publication of ENACT sparked controversy for several reasons, including a criticism that treatment of AS patients with ARPIs is active treatment (not AS), a lack of evidence for pathological progression being a valid surrogate endpoint, and high toxicity rates [37]. In all ARPI trials [32, 33, 34, 35, 36], side effects are observed, however, they appear to be short‐lived and associated with rapid recovery to normal function once treatment has stopped. As such, there is currently insufficient evidence to recommend ARPIs for AS patients.

## Non‐Hormonal Interventions

Alongside hormonal strategies, a range of non‐hormonal interventions has been investigated – repurposed metabolic and anti‐inflammatory agents, nutraceuticals, dietary and lifestyle modification – to minimise treatment toxicity while potentially modulating tumour biology or host factors (Table 2). Nutraceuticals – food‐derived compounds with purported medical or pharmaceutical benefits – have been of longstanding interest in PCa research [38]. For example, pomegranate extracts, rich in polyphenols, have been shown to inhibit AR expression and androgen signalling in PCa cells [39], while the antiproliferative effects of vitamin D and omega‐3 have been demonstrated in PCa murine models [40, 41].

**Table 2:** Summary of interventional clinical trials which investigated non‐hormonal agents as interventions in men with low‐ or intermediate‐risk prostate cancer on active surveillance.

Author/year	Study design	Number of patients	Population	Intervention	Primary endpoint	Follow‐up period	Conclusions
Marshall et al. [45] 2012	Prospective open‐label cohort study	44	Low‐risk PCa GS <6 PSA <10 ng/mL Clinical stage T1c or T2a	4000 IU vitamin D daily for 1 year	Biopsy after 1 year of treatment	12 months	55% improvement, defined as decrease in positive cores and no increase in GS at repeat biopsy
Thomas et al. [47] 2014	Prospective double‐blind placebo‐controlled randomised trial	199 AS (*n* = 121) WW following previous interventions (*n* = 78)	Histologically confirmed PCa AS or WW managed	Oral capsule TDS: Broccoli power 100 mg Turmeric powder 100 mg Pomegranate whole fruit power 100 mg Green tea 5:1 extract 100 mg equivalent	PSA level	6 months	Difference in median PSA rise between supplement group and placebo group 63.8% (*P* = 0.0008)
Bourke et al. [54] 2018	Prospective multi‐site phase II randomised controlled trial (PANTERA)	50	Low‐ or intermediate‐risk PCa on AS GS ≤3 + 4 Clinical stage ≤T2b Pre‐treatment PSA ≤20 ng/mL Life expectancy ≥10 years	Intervention (*n* = 25): aerobic exercise training (supervised and independent sessions) – 150 min exercise/week Control (*n* = 25): usual care with advice (Macmillan ‘Move More’ exercise education pack)	Trial acceptance rate (feasibility study) Progression to invasive treatment	12 months	Progression to invasive treatment over the 12‐month follow‐up period was equivocal (1 in intervention and 2 in control arm)
Kellogg Parsons et al. [52] 2020	Prospective randomised controlled trial (MEAL)	478	Age 50–80 years Biopsy‐proven PCa ISUP GG1 if <70 years old and GG2 if ≥70 years old Clinical stage ≤cT2a PSA <10 ng/mL	Intervention: Telephone counselling promoting consumption of seven or more daily vegetable servings (*n* = 237) Control group: written information about diet and PCa (*n* = 241)	Time to progression, defined as PSA level of 10 ng/mL or greater, PSA doubling time of < 3 years, or upgrading (defined as increase in tumour volume or grade) on follow‐up prostate biopsy	24 months	No significant differences in time to progression (adjusted HR 0.97, 95% CI 0.76–1.25)
Jarrard et al. [39] 2020	Prospective placebo‐controlled randomised trial	30	Organ‐confined, favourable‐risk PCa on AS	Pomegranate fruit extract 1000 mg once daily for 12 months	Serum and prostate tissue biomarkers such as PSA and IGF‐1 levels	12 months	No differences in IGF‐1 levels, PSA doubling time, or biopsy kinetics Reduced 8‐OHdG levels in prostate tissue
Dinneen et al. [46] 2022	Prospective double‐blind placebo‐controlled 3 × 2 factorial randomised trial (PROVENT)	94	Newly diagnosed low‐/favourable intermediate‐risk PCa: PSA <15 ng/mL ISUP GG <2 Biopsy core <10 mm maximum length Clinical stage <T2c	1: placebo 2: low‐dose (100 mg) aspirin 3: standard‐dose (300 mg) aspirin Patients randomised again to receive placebo or 4000 IU vitamin D (combination therapy)	Trial acceptance rate (feasibility study) Disease progression, defined as 50% increase in baseline PSA, new lesions on mpMRI, >33% volume increase in lesion size, histological upgrade, or 50% increase in maximum cancer core length	12 months	Disease progression 43.3% overall in the study population (specific treatment arms not reported)
Fleshner et al. [44] 2024	Prospective randomised double‐blind placebo‐controlled trial (MAST)	407	Biopsy‐proven low‐risk localised PCa on AS for < 1 year GS <6 Serum PSA <10 ng/mL prior to biopsy	Metformin 850 mg twice daily for 36 months	Time to progression, defined by time to primary therapy or pathological progression (>1/3 cores involved, >50% core involvement, Gleason pattern 4 or higher)	36 months	No difference in progression‐free survival observed between the two groups (*P* = 0.63)
Aronson et al. [51] 2025	Prospective randomised placebo‐controlled trial (CAPFISH‐3)	100	GS 3 + 4 or less Clinical stage ≤T2c PSA 25 ng/mL ISUP GG1 or GG2	Omega‐3 fatty acids (docosahexaenoic acid [DHA] and eicosapentaenoic acid [EPA]) 2.2 g once daily Dietary reduction <30% omega‐6 fatty acids (aiming for omega‐6 to omega‐3 ratio <4:1)	Change in Ki‐67 index from baseline to 1 year from same‐site biopsies compared between the groups	12 months	Significant difference in the change of Ki‐67 index between the groups (95% CI 2–52%, *P* = 0.043)

AS, active surveillance; GS, Gleason score; HR, hazard ratio; IGF‐1, insulin‐like growth factor‐1; ISUP GG, International Society of Urological Pathology Grade Group; mpMRI, multiparametric magnetic resonance imaging; PCa, prostate cancer; TDS, three times a day; WW, watchful waiting.

### Metformin

Based on chemopreventative and mortality risk reduction epidemiological data in cancers including PCa [42], metformin has been prospectively trialled as a chemoprotective agent in PCa [43]. The Metformin Active Surveillance Trial (MAST) was a phase III randomised double‐blind placebo‐controlled trial, which investigated the role of metformin in delaying time to disease progression over a 3‐year follow‐up period in 407 men with low‐risk PCa (Table S1) on AS [44]. Disease progression was defined as earliest occurrence of primary therapy (RP, RT, or hormonal therapy) or pathological progression (>1/3 of total cores involved, at least 50% of any one core involved, or Gleason pattern 4 or higher). There was no statistically significant difference in progression‐free survival between the two arms (*P* = 0.63) [44].

### Vitamin D and Aspirin

There have been two recent clinical trials exploring vitamin D supplementation in PCa patients on AS [45, 46]. A prospective open‐label cohort study in 44 patients with low‐risk PCa, who received daily supplementation with 4000 IU of vitamin D, reported at 1 year of follow‐up that 55% of men showed a decrease in the number of positive cores or a decreased GS, and 11% showed no changes at 1 year of follow‐up on repeat prostate biopsy [45]. The PROVENT feasibility RCT investigated aspirin with or without vitamin D in patients with low‐ or intermediate‐risk PCa on AS [46]. Of 94 assessable patients, 43.3% of patients had disease progression across treatment arms, but the authors did not compare progression rates between treatments as the primary outcome was trial recruitment. All single agents and combination regimens were well tolerated, with only two reported adverse events related to aspirin. Overall, there remains a paucity of evidence for vitamin D in men on AS and further large‐scale RCTs are required.

### Polyphenols

In a prospective cohort study of 30 men with localised PCa on AS, pomegranate fruit extract supplements reduced levels of 8‐OHdG (a marker of oxidative stress and DNA damage) in prostate tissue after 12 months of treatment [39]. The oral pomegranate extract‐containing capsule was well tolerated, with high compliance rates [39]. Various combinations of dietary supplements have also been trialled. The UK Pomi‐T study (Prostate Cancer and Polyphenols: Pomegranate, Green Tea, Broccoli and Turmeric Trial) examined the effect of a compound food supplement on PSA levels in 199 men with localised PCa [47]. A supplement containing 100 mg broccoli powder, 100 mg turmeric powder, 100 mg pomegranate powder, and 100 mg green tea extract was compared to a placebo. After 6 months, the median PSA rise was significantly lower in the Pomi‐T group than in the placebo group (14.7% vs 78.5%, respectively; *P* = 0.0008) [45]. However, PSA assessment has its shortfalls, and the primary endpoint did not incorporate other formal indicators of disease progression such as mpMRI or prostate biopsy. No difference between the groups in adverse events was reported [47]. Although not statistically significant, there was a trend suggesting improved urinary flow, prostatic symptoms, and bowel function in the supplement group [47].

### Folic Acid

Some controversy exists over the effect of folic acid in PCa, specifically the hypothesis that higher blood folate levels may be associated with an increased risk of PCa based on case–control studies [48]. A nested case–control study of 6000 men, matched on age of patient at time of serum sampling and date of serum sample taken, reported a weak positive association between serum folate concentration and PCa risk (highest vs lowest quintile odds ratio 1.15, 95% CI 1.07–1.84; *P* = 0.04) [49]. These results should be interpreted with caution, however, given that the variation in folate levels among different populations and countries did not reflect local PCa incidence and may be attributable to other observed differences and confounding factors [49].

### Diet and Omega‐3 Supplementation

In a cross‐sectional study of 157 men (nested within a phase II trial) with low‐risk PCa on AS, higher long‐chain omega‐3 eicosapentaenoic acid (LCω3‐EPA) levels in prostate tissue were associated with significantly lower odds of high‐grade disease at repeat biopsy (odds ratio 0.25, 95% CI 0.08–0.79; *P* = 0.03) [50]. In a phase II RCT of 100 men with localised PCa on AS, a high omega‐3, low omega‐6 diet with fish oil supplementation for 1 year significantly reduced tumour proliferation in same‐site prostate biopsies, measured by the Ki‐67 proliferative index, compared with a control diet [51]. Ki‐67 decreased by ~15% in the diet and fish oil group and increased by ~24% in controls (*P* = 0.043), while no significant differences were observed for Gleason Grade Group, tumour length, Decipher score, or PSA. Four participants in the diet and fish oil group withdrew due to fish oil‐related gastro‐intestinal adverse events.

The Men's Eating and Living (MEAL) RCT enrolled 478 men with CPG1 PCa on AS to assess whether a telephone‐based counselling programme promoting high vegetable intake reduced PCa progression compared with written dietary information [52]. Over 24 months, 245 progression events occurred, with no significant difference in time to progression between the intervention and control groups (adjusted HR 0.97, 95% CI 0.76–1.25; *P* = 0.76). In the Canary Prostate Active Surveillance Study (Canary PASS) of 564 men with localised PCa on AS, questionnaire‐based assessment of healthy diet adherence was performed at enrolment and disease reclassification was assessed over a median 7.8‐year follow‐up [53]. Among 237 men who experienced upgrading, higher adherence to dietary patterns was not associated with reduced progression risk [53]. Taken together, these findings do not support increased vegetable consumption or healthy diet adherence as an effective strategy to slow PCa progression. Nonetheless, given the benefits for prevention of other chronic diseases, these diets are a prudent choice for men on AS.

### Exercise

The Prostate Cancer Novel Therapy (PANTERA) trial explored exercise training with behavioural support for patients with low‐ and intermediate‐risk PCa managed by AS [54]. This feasibility study randomised 50 men: 25 to exercise training and 25 to usual care. At 12 months, patients randomised to exercise training had reduced body mass (mean reduction 2.0 kg, 95% CI 1.1–2.9), reduced systolic (mean pressure 13 mmHg, 95% CI 7–19) and diastolic blood pressure (mean pressure 8 mmHg, 95% CI 5–12), and improved quality of life as assessed by EuroQol 5 Dimensions (EQ‐5D; mean score = 13, 95% CI 7–18). Three patients progressed to radical treatment (one on exercise training and two on usual care).

In summary, non‐hormonal interventions for men on AS have shown signals of benefit in small studies, such as improvements in biomarkers, PSA kinetics, and quality‐of‐life measures. However, the evidence base is constrained by small sample sizes, heterogeneous study designs, and a lack of adequately powered, large‐scale RCTs with clinically meaningful endpoints. While these approaches are generally well tolerated, the current data are insufficient to support their routine use in clinical practice.

## Imaging and Molecular Biomarkers

Baseline mpMRI has prognostic value for patients on AS and is increasingly used for disease monitoring [55], although the optimal follow‐up schedule remains debated [56, 57]. The Prostate Cancer Radiological Estimation of Change in Sequential Evaluation (PRECISE) criteria, developed by international consensus, standardise reporting of MRI changes during AS [6], and were recently updated to distinguish stable findings in visible vs non‐visible disease, given the higher progression risk to Gleason 4 + 3, and to treatment in MRI‐visible cancers [14]. Despite this, a systematic review of 375 AS protocols found mpMRI was rarely incorporated: only 9.1% used it for monitoring, while 72% mandated repeat surveillance biopsies, and 87% used histological upgrading for reclassification [58]. Most studies of hormonal therapy for AS also used biopsy‐based histological upgrading as the sole progression endpoint. An expert consensus, however, now recommends omitting routine biopsies in patients with stable mpMRI and using combined mpMRI and PSA monitoring to determine further biopsy or change in management [57]. Further standardisation of the progression criteria for AS incorporating mpMRI is needed before these approaches can be fully adopted in practice.

Tissue‐based molecular assays may complement PSA, histology, and mpMRI in AS risk stratification and monitoring. Decipher (Veracyte), Oncotype Dx (Exact Sciences), and Prolaris CCP (Myriad Genetics) are mRNA‐based gene expression classifiers, while ProMark is protein‐based. Of these, only Prolaris has been validated for risk stratification and treatment decision‐making in both UK and US populations [59, 60]. In a cohort of 19 215 men, the combination of Prolaris with clinical data to generate a prognostic score increased AS eligibility from 42.6% (based on clinicopathological features alone) to 68.8% [60], and in a cohort of 103 cases, it reduced treatment change from interventional to non‐interventional by 37.2% [59]. Decipher scores performed on prostate biopsies of the ENACT trial cohort at trial screening, and at 1 and 2 years were positively associated with pathological or therapeutic progression in the AS arm (HR 1.17, 95% CI 1.01–1.35; *P* = 0.04) and showed a nonsignificant trend in the enzalutamide arm (HR 1.50, 95% CI 0.91–2.48; *P* = 0.11) [61]. Molecular biomarkers may help to refine patients’ eligibility for AS, particularly for CPG2 disease or those with indeterminate mpMRI findings.

## Discussion

Reducing disease progression risk for PCa patients on AS remains an important unmet clinical need. A major challenge is the lack of strong consensus among international guidelines on AS management, including eligibility, monitoring protocols (including mpMRI), and definitions of progression. Repeat sampling could result in histological disease upgrading in up to 35% of cases [56]. Several ongoing trials should provide some clarity on the role of mpMRI as a biomarker in AS monitoring [28, 35, 55]. The recent international consensus recommends that a rising PSA level should prompt an MRI rather than a biopsy or discussion of treatment, reserving biopsy discussion for MRI changes or increased PSA density [57].

Studies investigating hormonal and non‐hormonal interventions have included patients with a range of baseline profiles, and different criteria used to define that risk. Whilst all agree that the presence of Gleason pattern 4 confers higher progression risk, MRI visibility is emerging as an additional biomarker for disease progression. The role of PSA density (at baseline, and during surveillance), and size of tumour – whether defined by maximum cancer core length or MRI lesion size – are also being explored for their association with disease progression.

The heterogeneity of study design and clinical management in AS trials limits direct comparisons among strategies. Pharmacological therapies may offer the greatest short‐term benefit in patients with higher progression risk, whereas nutraceuticals, diet and exercise may have longer‐term benefits in lower‐risk disease. Whilst 5ARIs and ARPIs remain controversial due to concerns around toxicity and oncological safety, as well as proven mortality benefit, studies have not reported high rates of attrition secondary to adverse events. It is also imperative to minimise toxicity and maintain quality of life in AS patients who are otherwise asymptomatic from their disease. Therefore, well‐powered ongoing trials are timely and are warranted, especially as more patients – particularly those with intermediate‐risk features – are choosing AS. Future research should integrate biomarker profiling with standard AS protocols, coupled with long‐term outcomes data, to refine patient selection and surveillance strategies.

## Disclosure of Interests

S.G. is a fully paid employee of Johnson and Johnson Ltd. B.L. receives honoraria for public speaking from Parsek UK Ltd, consultancy fees from Digital Surgery Ltd, MDOutlook, and honoraria from AstraZeneca PLC and Astellas Pharma Europe Ltd. C.M.M. receives research funding from SpectraCure, has received speaker fees from Bayer, Ipsen and Janssen and conference support and fees for proctoring international surgeons in high‐intensity focused ultrasonography (HIFU) from SonaCare. J.W.F.C. has received reimbursement for consultancy from AstraZeneca, Ferring, Roche and Janssen, speaker fees from Bristol Myers Squibb, Merck Sharp & Dohme, Janssen, Astellas, Pfizer and Roche, honoraria for membership in advisory boards from Ferring, Roche, Bristol Myers Squibb and Janssen, and research funding from Roche. He is the Chief Investigator of the FINESSE trial [29] and an unpaid trustee Weston Park Cancer Charity. These organisations did not have any role in the writing of this manuscript. P.D.S. receives honoraria for membership of GRAIL's scientific advisory board and funding for the FINESSE trial [29] and for a clinical trials unit specialising in cancer prevention. The other authors declare no conflicts of interest.

## Funding

B.W.L. is funded by the North East London and East of England Cancer Alliances and NHS England. C.M.M. is funded by an National Institute for Health and Care Research (NIHR) Research Professorship, the Medical Research Council, Cancer Research UK, Prostate Cancer UK, Movember and the Bob Willis Fund. J.W.F.C. is funded by an NIHR Research Professorship. P.R. is funded by Barts Charity (MGU0596, G‐002724), Prostate Cancer UK (RIA22‐ST2‐001), and the North East London Cancer Alliance.

## Supporting information


**Table S1.** Comparison of patient characteristics between the three‐tiered NICE risk stratification model and Cambridge Prognostic Group model.
