# Supplementary material for: Interventions for patients with prostate cancer on active surveillance: a narrative review
Source: BJU Int. 2025 Oct 6;136(6):990–1001. doi: 10.1111/bju.70005 (PMC12606547; doi:10.1111/bju.70005)
Supplement: Supplementary file 1 — Table S1. Comparison of patient characteristics between the three‐tiered NICE risk stratification model and Cambridge Prognostic Group model. [file BJU-136-990-s001.docx]

**Supplementary Table 1.** **Comparison of patient characteristics between the three-tiered NICE risk stratification model and Cambridge Prognostic Group model**

| **D’Amico risk stratification based on treatment failure** | | **NICE: CPG risk stratification based on prostate cancer mortality** | |
| --- | --- | --- | --- |
| **Group** | **Patient characteristics** | **Group** | **Patient characteristics** |
| Low-risk | GS <6  and  PSA < 10 ng/ml  and  stage T1-T2a | 1 | GS 6 (GG 1)  and  PSA < 10 ng/ml  and  stages T1-T2 |
| Intermediate-risk | GS 7  or  PSA 10-20 ng/ml  or  stage T2b  and nothing higher | 2 (favourable) | GS 3+4 = 7 (GG 2) and PSA <10  or  GG1 and PSA 10-20 ng/ml and stages T1-T2 |
|  |  | 3 (unfavourable) | GS 3+4 = 7 (GG 2) and PSA 10-20 ng/ml and stages T1-T2  or  GS 4+3 = 7 (GG 3) and stages T1-T2 |
| High-risk | PSA > 20 ng/mL  or  GS > 7  or  stage T2c    Locally advanced disease: any PSA, any GS, stage T3-4 or lymph node positive disease | 4 | GS 8 (GG4)  or  PSA > 20 ng/mL  or  stage T3 |
|  |  | 5 | Two or more of the following: GS 8 (GG4), PSA > 20ng/mL, stage T3  or  GS 9 or 10 (GG5)  or  stage T4 |

*NICE=National Institute for Health and Care Excellence; CPG=Cambridge Prognostic Group; GS=Gleason score; PSA=prostate specific antigen; GG=Grade Group.*
